# Looking through the Lens: The Reality of Telesurgical Support with Interactive Technology Using Microsoft's HoloLens 2

**DOI:** 10.1155/2022/5766340

**Published:** 2022-03-01

**Authors:** Kees van der Putten, Mike B. Anderson, Rutger C. van Geenen

**Affiliations:** ^1^Zimmer Biomet Neth. B.V., Netherlands; ^2^Zimmer Biomet, Warsaw, IN, USA; ^3^Department of Orthopaedic Surgery, Amphia Hospital, Breda, Netherlands

## Abstract

Reality technologies in the orthopaedic arena have been increasing in use over the last decade, including virtual reality (VR), augmented reality (AR), and mixed reality (MR). MR is one of the most recent innovations and perhaps the most promising for improving the overall surgical experience. The purpose of this case report was to demonstrate a complex total knee arthroplasty case where unplanned remote assistance was used for telesurgical support using the HoloLens 2.

## 1. Introduction

**Figure 1:**
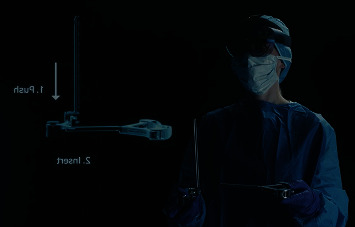
The HoloLens 2 mixed-reality headset is used in visual instrumentation setup in this example of using a holographic display. Used with permission from Zimmer Biomet, Inc.

Reality technologies in the orthopaedic arena have been increasing in use over the last decade [1]. These have included virtual reality (VR), augmented reality (AR), and mixed reality (MR). MR is one of the most recent innovations and perhaps the most promising for improving the overall surgical experience. This is primarily due to its ability to allow interactions with holographic images placed in the real world via a mixed-reality headset (Figure 1). Prior reality technology has been limited to virtual settings or unresponsive holographs. However, these technologies have demonstrated value in both surgeon training and remote assistance [1, 2]. Thus, it is reasonable that interactive reality technologies are the next progression in advanced surgeon training [3, 4], intraoperative guidance [5], and remote assistance or telesurgical support [2].

Greenfield et al. [2] have previously demonstrated the successful use of AR in providing remote surgical assistance associated with a traumatic hand injury. This case report was valuable in demonstrating a proof of concept (POC) for telesurgery where surgeon specialists can interact with the surgical team for mentorship and surgical decision-making. The authors reported that the limited resources and expertise at the surgical site were compensated for by using a remote surgeon specialist interacting via remote assistance. This case report has proven valuable for demonstrating the benefits of adopting reality technologies.

Recently, a POC team was created by a medical device manufacturer (Zimmer Biomet the Netherlands) to demonstrate the feasibility of providing remote telesurgical support with the HoloLens 2 head-mounted lens and the Microsoft Dynamics 365 Remote Assist software (Microsoft, WA, USA) in total knee arthroplasty (TKA). The Dutch POC team consisted of an orthopaedic surgeon working in two different locations, the manufacturer's account manager, and a POC lead from the manufacturer.

The purpose of this case report is to demonstrate a complicated TKA case where unplanned remote assistance was used for telesurgical support using the HoloLens 2.

## 2. Ethical Considerations

This is a single case report on the ability to use a mixed-reality headset with remote assistance software to provide telesurgical support between an industry representative and an orthopaedic surgeon. The report is anonymized per ICMJE standards, removing all identifying details associated with patient information. As such, informed consent from the patient and review by the local ethical committee were not required. Additionally, all images used as examples of the HoloLens application have been altered to avoid the identification of those in the images.

Privacy is of the utmost concern when it comes to both patients and healthcare professionals. The HoloLens 2 Privacy and Data Protection information notes that the device contains both network and operating system securities with data protection [6]. The data protection includes the use of Azure integration which encrypts the data traveling through the cloud with the use of transport layer security. Azure documentation demonstrates compliance with ISO/IEC 27001, 27017, and 27018 amongst others. Local memory is also protected by encryption, and the program requires personal login and passwords.

## 3. Telesurgical Support with HoloLens 2 Case Report

**Figure 2:**
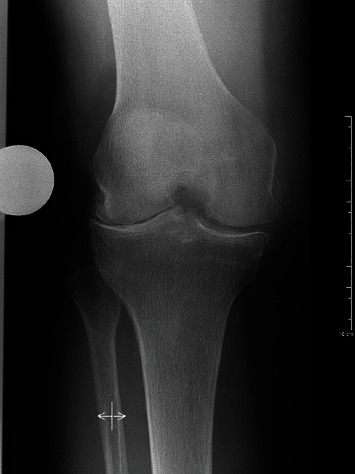
Preoperative radiographs showing the anteroposterior view of the right knee.

**Figure 3:**
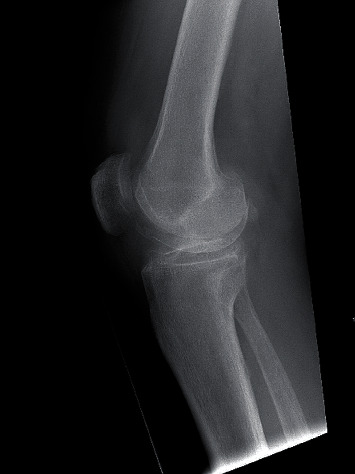
Preoperative radiographs showing the lateral view of the right knee.

**Figure 4:**
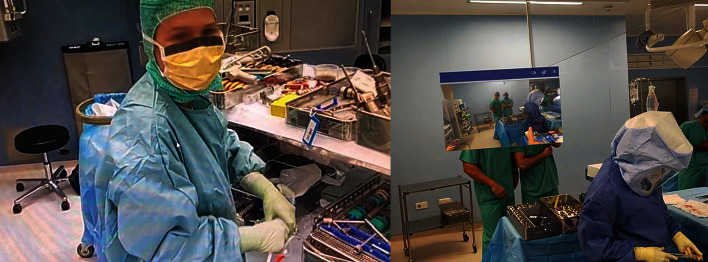
Examples of the product manager's view of a remote operating room through the HoloLens worn by the surgeon.

During what was expected to be a standard TKA for a 78-year-old female patient with lateral compartment osteoarthritis of the right knee (Figures 2 and 3), the surgeon in training was confronted with an iatrogenic complete lateral ligament transection that required additional constraint. Considering the age of the patient, the supervising surgeon chose a rotating hinge prosthesis (NexGen® Rotating Hinge Knee, Zimmer Biomet, Warsaw, IN, USA) to achieve immediate weight bearing and to avoid elongated rehab associated with ligament reconstructions. Given the recent introduction to the hospital and surgeon, of this knee implant system, additional support and guidance was needed intraoperatively. Unfortunately, this specific case was unexpected and onsite support was not available. Further, the available implant product manager (PM) was located more than 100 kilometres away. Given the recent development of the POC team to demonstrate the utilization of remote assistance with MR, this case presented the opportunity to use the technology in a real-world emergent telesurgical support experience. It was determined that the implant PM, who was not part of the original POC team and was unfamiliar with the MR device, would provide this support. The HoloLens 2 MR headset (Microsoft, WA, USA) is a wireless holographic computer contained within a wearable headset [7]. It features a holographic lens which contains sensors and displays. Head and eye tracking is performed with light and infrared cameras, and movement is assessed using sensors (accelerometers, gyroscope, and magnetometers). The Microsoft Dynamics 365 Remote Assist software with HoloLens 2 (Microsoft, WA, USA) was used. It is an advanced video communication tool that enables hands-free collaboration with remote experts in real time. In wearing a HoloLens 2, the user can share a real-time view of the environment with remote collaborators eliminating the need for them to be on site (Figure 4). With tools such as annotation, ability to move virtual documents to the real world, and ability to deploy across multiple devices, these advanced features enable more effective on-demand collaboration. Further, the telesurgical setting allows for mobile, real-time, intraoperative views.

### 3.1. Setup

Though the PM was unfamiliar with the MR technology, the urgency of the situation and the distance (>100 kilometres) separating the surgeon with the product team required innovation. Thus, the surgeon and PM agreed to proceed with the telesurgical support using the MR device. This resulted in an estimated two hours of time saved for the case as the PM was no longer required to travel to the facility to provide support.

**Figure 5:**
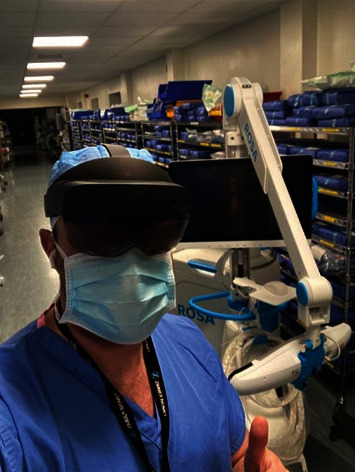
Example of the HoloLens 2 in use by a product manager at a distant location.

First, the PM was provided credentials by the POC team and instructed on the use of remote assistance with the MR device. The PM placed the lens on (Figure 5) and completed the login process to the software to remotely connect with the surgeon. The surgeon, also wearing a lens, and PM then connected via the remote assist application. This allowed for direct visualization of the operating theatre by the PM through the surgeon's lens (Figure 4). With the use of both lenses, there was no need for additional camera placement or hand-held interfaces, despite the geographic barriers as well as a prompt response to an urgent situation. The actual time required to set up the credentials and establish the link was minimal in comparison to waiting for a local representative, or distant PM, to arrive at the operating theatre. Additionally, the heads-on view provided direct line of sight for the PM to see exactly what the surgeon was viewing. This allowed clear and instant visualization of the operating field per the surgeon's perspective throughout the procedure, including the instrument table.

### 3.2. Procedural Support

The initial planning of the case included a standard parapatellar medial approach without the use of a tourniquet. As previously noted, a complete iatrogenic lateral ligament transection occurred, and it was determined that the rotating hinge prosthesis was needed.

Upon establishing the connection between MR headset, the PM and surgeon began discussion on the critical information needed to support the case. This included discussing each step of the procedure with the surgeon and guiding the OR staff in assembling the instruments and the prostheses. The visual and auditory connection provided clear and transparent communication when looking through the lens of the MR headset without the need for the PM to be present in the theatre.

Problems were solved in real time, including walking through an improvised method of instrumentation. This was due to one tray becoming unsterile in the hustle to get the case done, which would normally have been prepared prior to incision. The PM used the MR headset to directly communicate the walk-around during the procedure. This allowed the surgeon to stay focused on the task at hand and successfully perform the procedure using the rotating hinge implant. There were no further intraoperative complications aside from the initial ligament transection. The senior author reports no other interruptions in the surgical workflow with the use of the MR headset.

**Figure 6:**
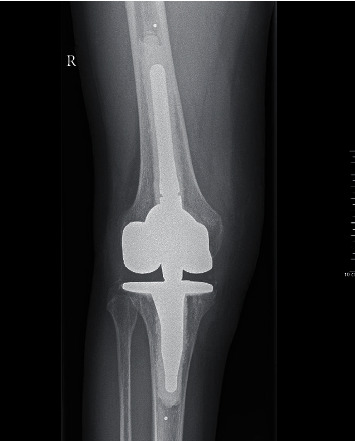
Postoperative radiographs showing the anteroposterior view of the right knee.

**Figure 7:**
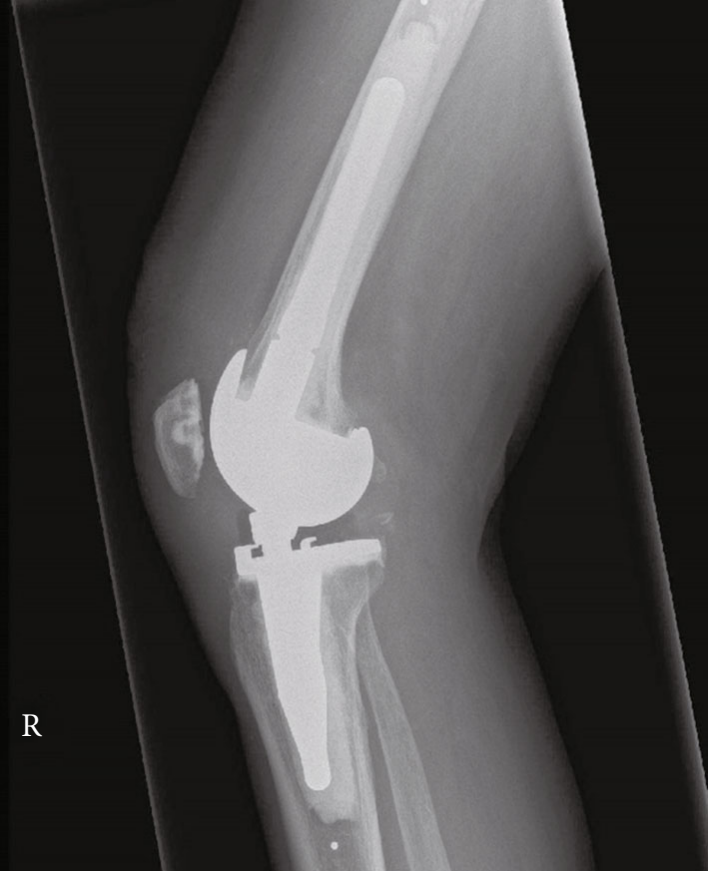
Postoperative radiographs showing the lateral view of the right knee.

The surgeon was satisfied with the final component positioning (Figures 6 and 7, postop X-rays), and the surgical team continued with the closure of the procedure.

At the last follow-up, the patient has recovered well with no additional complications.

## 4. Discussion

This case demonstrates the relatively nonexistent learning curve of the industry PM to utilize remote assist via a MR headset for telesurgical support with the HoloLens 2 interactive technology. Given the current environment associated with the COVID-19 pandemic, including travel restrictions and limited personnel, the ability to provide prompt telesurgical support from any location may improve emergent surgical team support by providing industry subject matter experts regardless of proximity.

Consistent with augmented reality, MR provides the users the ability to communicate with real-time auditory and visual connection. What sets MR aside is its ability to use interactive holographic projections that align with the real world. In a recent review, Verhey et al. [1] suggested that MR may be the most disruptive of the reality technologies, given the potential for surgeons to interact with imaging and patient information within the operating field. This is supported by the recent case report of Gregory et al. [8] who described the use of a HoloLens headset combined with computer navigation in performing a total shoulder arthroplasty. Not only was mixed reality used to manipulate in-field holographs, but a collaborative mode was used to share the field of vision with surgeons across the globe. This supports the notion that not only can MR assist the surgeon intraoperatively with holographic displays, but it can also provide immersive collaboration, with both surgeons and industry representatives.

Unfortunately, given the novelty of this system and the immediate need for remote assistance, holographic aids were not used in this case. This is perhaps a limitation of the current application; however, applications are being released that include holographic recognition of surgical instruments and holographic screens for other intraoperative digital technologies. Other technologies, such as video conferencing systems, have been shown to be valuable in remote surgical education across the globe, including broadcasting of surgical procedures [9, 10]. This often requires the use of extensive operating theatre upgrades for catching different camera angles, additional microphones, and even a surgeon narrator. Madani et al. [9] noted limitations of such systems for open procedures given the limited mobility of the cameras, though they did suggest the ability to use head-mounted cameras may resolve these concerns. Morimoto et al. [10] describe the use of “action cameras” to record surgery from the surgeon's perspective, but note that an ideal camera is not currently available. Though these recordings may perform well in educational settings, they do not allow real-time interaction from the surgeon's viewpoint intraoperatively. Further, the security measures for these “action cameras” appear limited in comparison to certified methods reported by Microsoft [6, 11, 12]. Head-mounted displays, such as the HoloLens 2, may provide more realistic and immersive education opportunities for surgical trainees [10]. The MR headset used in this case report combines the multiple products used in a modern video conferencing capable operating theatre into a smart headset with sensors, cameras, and microphone built in, and there is no need for a surgeon narrator. This system is not limited to specific operating theatres and provides users the ability to remotely connect and immediately immerse themselves in the situation.

This case study demonstrates the ability to use the HoloLens 2 with Microsoft Dynamics 365 Remote Assist Software to provide immediate telesurgical support by an industry representative, the PM, to the operating surgeon approximately 100 km apart. The data is transferred via an encrypted and secured cloud-based program and provided the PM a real-time surgeon viewpoint of the operating theatre, including direct line of site to the operative knee, the operating instruments, and even the surgeon's hands.

Adoption of disruptive technologies is not just a future vision but is occurring in all aspects of the surgical experience today. As surgeons and industry collaborate to improve patient care and move toward creating access to a greater knowledge base, the orthopaedic surgical world is likely to see improvements in the delivery of patient care.

## Data Availability

This is a single case report and all available data are in the manuscript.
